# Differential Sampling of AC Waveforms Based on a Commercial Digital-to-Analog Converter for Reference

**DOI:** 10.3390/s24072228

**Published:** 2024-03-30

**Authors:** Yanping Wang, Xiaogang Sun, Jianting Zhao, Kunli Zhou, Yunfeng Lu, Jifeng Qu, Pengcheng Hu, Qing He

**Affiliations:** 1 School of Instrumentation Science and Engineering, Harbin Institute of Technology, Harbin 150080, China; 18b901028@stu.hit.edu.cn (Y.W.); hupc@hit.edu.cn (P.H.); 2 National Institute of Metrology, Beijing 100029, China; zhoukl@nim.ac.cn (K.Z.); luyf@nim.ac.cn (Y.L.); qujf@nim.ac.cn (J.Q.); heqing@nim.ac.cn (Q.H.)

**Keywords:** digital-to-analog converter, differential voltage, precision measurement, uncertainty

## Abstract

This paper introduces an innovative differential sampling technique for calibrating AC waveforms, leveraging a commercially available 16-bit digital-to-analog converter (DAC) as the reference standard. The novelty of this approach lies in its enhanced stability over traditional direct sampling methods, especially as the frequency of the AC waveform increases. Notably, this technique provides a cost-effective sampler alternative to the differential sampling methods that rely on a programmable Josephson voltage standard (PJVS). A critical aspect of this methodology is the precise measurement of the DAC’s output voltage, for which a static measurement strategy is adopted to utilize the exceptional linearity and transfer accuracy of the Keysight 3458A (Santa Rosa, CA, USA) in its standard DCV mode. The differential sampling method has demonstrated good accuracy, achieving a near 1 µV/V agreement with a pulse-driven AC Josephson voltage standard (ACJVS) across a 40 Hz to 200 Hz frequency range. The method attained an expanded uncertainty (*k* = 2) of 1 part in 10^6^ while measuring a 0.707107 V_RMS_ sine wave at 50 Hz, showcasing its efficacy in precise AC waveform calibration.

## 1. Introduction

The calibration of AC signals is conventionally conducted using a thermal converter (TC) [1]. However, this method only reflects a root mean square (RMS) amplitude and cannot reflect the spectral information of the calibrated AC voltage [2]. In addition, this technique requires multiple ac–dc comparison steps and the output voltage of the thermal converter in the AC case will be different from the DC case [3]. In recent years, differential sampling based on a PJVS as a reference for measuring AC signals has been widely used in national metrology institutions (NMIs) [4,5,6,7]. Using the quantized step of the stepwise approximated waveforms generated by PJVS, the differential sampling method can accurately measure the unknown AC voltage with the transients discarded [8,9,10,11,12,13]. However, the PJVS system requires liquid helium for operation, and AC voltage calibration with it is complex and costly [8]. Therefore, differential sampling measurements of AC signals based on a PJVS can only be conducted in NMIs. The primary method for daily measurements is still to directly measure the AC signals with commercial instruments, e.g., a Keysight 3458A voltmeter. Unfortunately, the accuracy of direct measurement for AC signals is relatively low [14].

The Keysight 3458A is well known for its superior linearity and transfer accuracy in standard DCV mode [13]. To make the most of its excellent performance in this mode and reduce gain deviations when measuring AC signals, this paper proposes a differential sampling method that uses a commercial DAC (which is in one channel of the NI 6733) as a reference to determine the amplitude and phase of AC waveforms. The main contributions of this paper can be listed as follows:

(1) A differential sampling system with a commercial DAC for reference has been developed. A combined uncertainty of 1 part in 10^6^ (*k* = 2) was archived with a frequency of 50 Hz and an amplitude of 1 V.
(2) Large AC signal measurements are converted into large DC signal measurements and small AC signal measurements. The Keysight 3458A is excellent for DC voltage measurement. The experiment proves that measuring small voltages provides greater accuracy than large voltages with the same aperture time, making this differential sampling method more stable and accurate than direct sampling measurement.
(3) The reference voltage is determined through a static measurement method rather than dynamically sampled, leading to a notable enhancement in the stability and precision of measurements.
(4) The uncertainty propagation model of DFT is derived, which is quite important for measurement procedure design and the uncertainty estimation of the final result.

## 2. Accurate Measurement of Reference Voltage

When using a commercial DAC to produce stepwise approximated waveforms as a reference for measuring AC waveforms through differential sampling, it is crucial to measure the reference voltage accurately. In this section, a static measurement method is proposed for measuring the reference voltage. Furthermore, this section analyzes the drift of the DAC and discusses methods for minimizing effects to enhance the accuracy of reference voltage measurements. 

**Figure 1:**
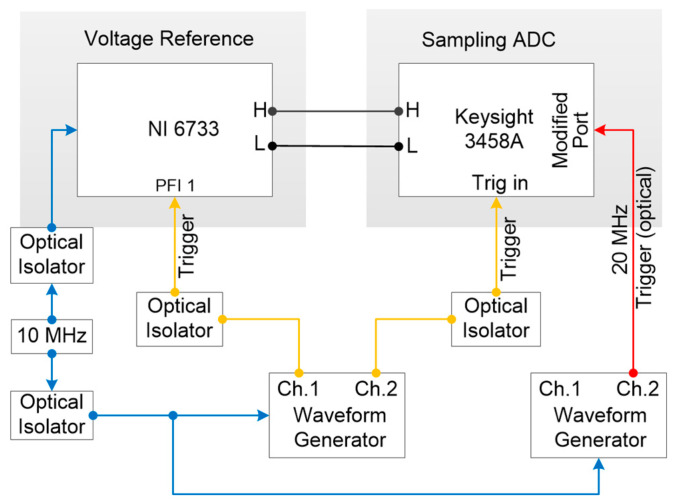
Block diagram of the measurement configuration for reference voltage.

Figure 1 is a block diagram of the measurement configuration for the reference step wave. The Keysight 3458A was modified to use a 20 MHz and 5 V_pp_ sinusoidal signal which is provided by the signal generator (Keysight 33522A) as an external clock signal. The trigger signals of the DAC and sampling voltmeter are both provided by another signal generator. The external reference clock of the signal generators is provided by the same 10 MHz frequency reference to realize the synchronization between all signals. All clock signals and trigger signals are connected by the self-made optical coupling isolator to eliminate the influence of ground noise on the measurement results.

### 2.1. A Static Measurement Method to Measure Reference Voltage

**Figure 2:**
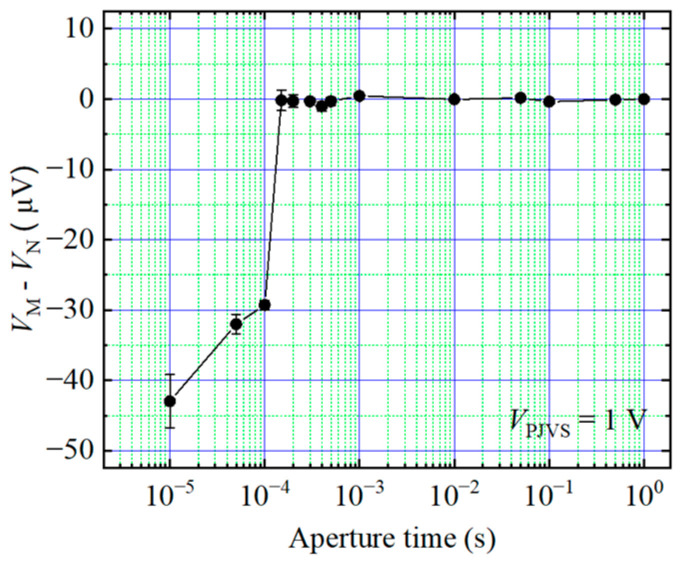
Difference voltage between the measured DC voltage (*V*_M_) and the nominal voltage (*V*_N_) at different aperture times. The nominal voltage of the calibrator is 1 V.

Accurately sampling the reference voltage in a dynamic system is a challenging task. As the frequency increases, the aperture time of the sampler for each reference step decreases. This shorter aperture time leads to an increased error of the voltmeter, which is shown in Figure 2. Therefore, in this paper, the measurement of AC waveforms is divided into two parts: the differential sampling process and the reference voltage calibrating process. In the differential sampling stage, the DAC outputs reference stepwise waveform, and the differential voltage between it and the AC waveform is measured by the sampler. In the reference voltage calibration stage, DAC outputs the DC waveforms of each step, and the step voltages are measured by the sampler sequentially. The reference voltage calibration stage is a static measurement process which provides higher calibration accuracy due to a longer sampler aperture time. The differential sampling stage is a dynamic measurement process, but the error from the sampler has little influence on the small differential voltages. The reference waveform calibration process is periodically performed with the period determined by the DAC drift, which is discussed in the following section.

**Figure 3:**
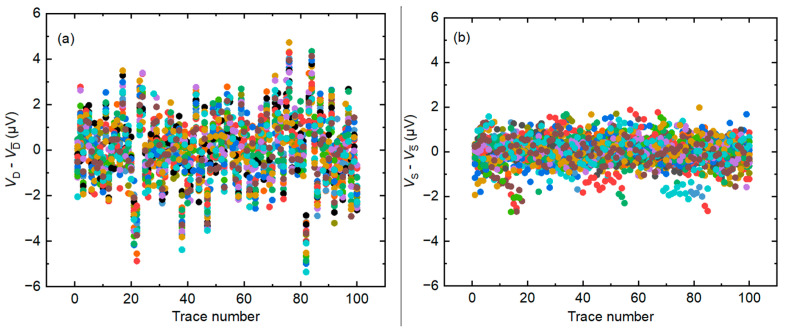
Deviation of each step measured with the two-measurement method (**a**) with the dynamic measurement method and (**b**) with the static measurement method. The steps are represented by different colors, with *N* = 20.

Figure 3a shows the deviation of each step measured with the dynamic measuring method, where the voltages are measured when the DAC outputs stepwise waveforms, and Figure 3b displays the deviation of each step measured with the static measuring method, where the voltages are measured when the DAC outputs DC waveforms. *V*_D_ is the voltage measured with the dynamic method and $V_{\bar{D}}$ is the average of *V*_D_, *V*_S_ is the voltage measured with the static method and $V_{\bar{S}}$ is the average of V_S_. The amplitude of the reference waveform is 1 V and the frequency is 50 Hz with *N* = 20, where *N* is the number of DAC waveform steps. Both measurement methods are measured in the DCV mode of the sampler and the voltage range of the sampler is 1 V. The aperture time is 1 s for static measurement and 400 µs for dynamic measurement. It is important to ensure that the total aperture time per step is equal between the two measurement methods. Figure 3 shows a higher deviation in dynamic measurements, indicating that static measurements are more stable and require less time.

**Figure 4:**
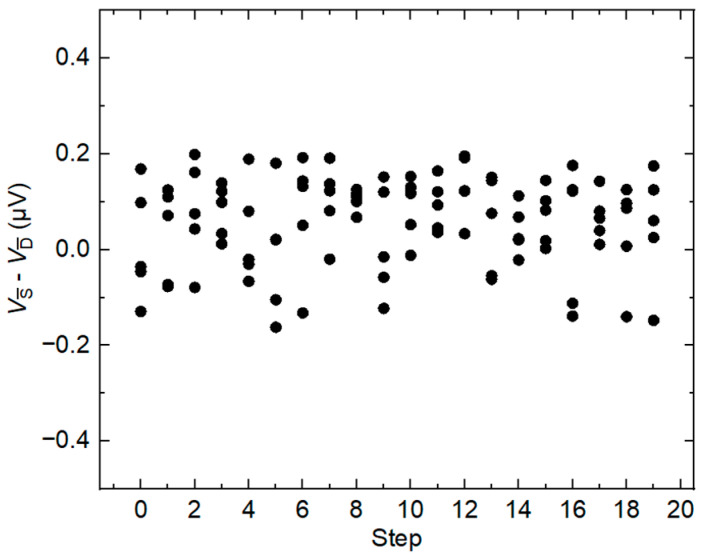
The voltage difference between the dynamic measurement method and the static measurement method.

Since differential sampling is a dynamic process, it is necessary to ensure that the DAC step voltage measured in static mode is the same as the step voltage in dynamic mode. To verify this, the difference voltage is measured between two DAC channels and a dynamic stepwise approximated waveform is generated in one channel (channel A), while the other channel (channel B) generates DC waveforms that correspond to each step in channel A. The voltage difference between the two channels falls within the acceptable range (less than 0.2 µV), as shown in Figure 4. This suggests that measuring the reference voltage using a static method is a viable alternative to the dynamic method. However, as discussed previously, the advantage of the static method will become more evident as frequency increases.

### 2.2. Drift of the DAC Output

**Figure 5:**
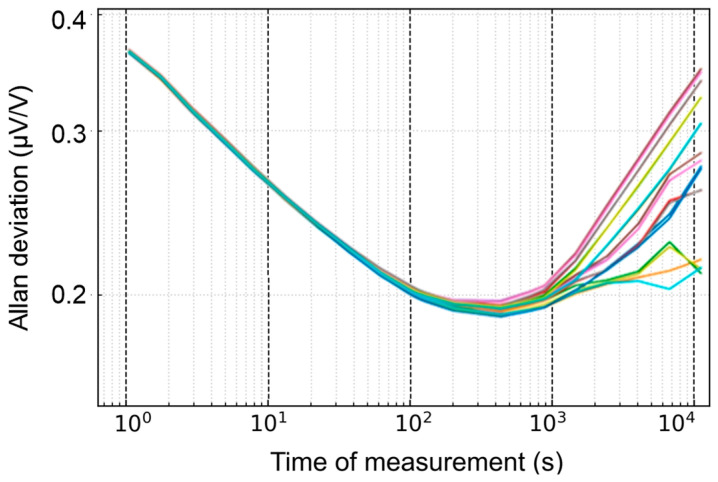
Allan deviation analysis of each step within 12 h (to depict the trend clearly, only three hours were displayed). The steps are represented by different colors, with *N* = 20.

Compared to the PJVS system, the step voltage of a commercial DAC drifts with time. As depicted in Figure 5, the Allan deviation analysis [15] enables us to determine each step’s long-term stability and minimum standard deviation of the reference voltage. The amplitude of the reference waveform is 1 V and the frequency is 50 Hz with *N* = 20. The voltage range of the sampling voltmeter in the experiments is 1 V. Each step of the reference waveform is provided by the DAC in sequence, and the sampler obtains them by the static measurement method mentioned in the previous section. During the measurement at 200 s, the noise is reduced to below 2 parts in 10^7^, but it increases rapidly at around 1000 s. 

**Figure 6:**
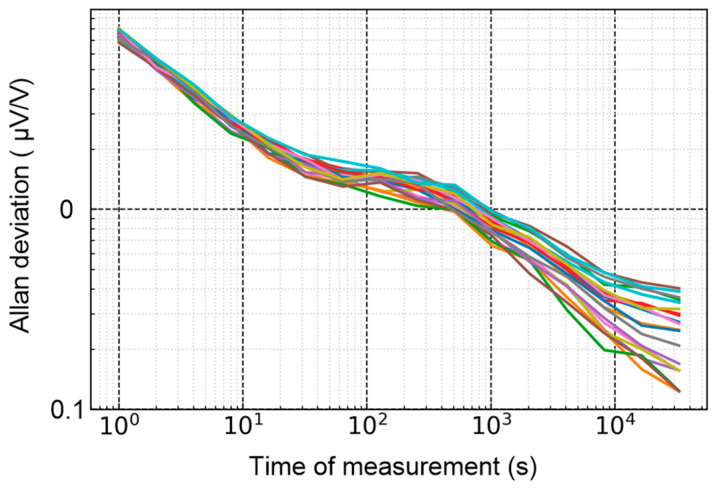
Allan deviation analysis of the voltage difference between the dynamic method and the static method within 12 h. The steps are represented by different colors, with *N* = 20.

Additionally, to determine if the DAC’s dynamic stepwise approximated waveform aligns with the long-term drift of the static DC waveform, an Allan deviation analysis of the voltage difference between the dynamic and static voltage is conducted over 12 h. One of the channels in the DAC (channel A) provides a stepwise approximated waveform (*N* = 20) with a frequency of 50 Hz and an amplitude of 1 V, and the other channel (channel B) generates DC waveforms that correspond to each step in channel A. The voltage difference between the two channels is obtained by the sampler. As shown in Figure 6, the long-term drift of the dynamic voltage is consistent with the static voltage.

Therefore, to achieve optimal measurement results, the reference voltage should be calibrated by static measurement within 1000 s to minimize the influence of reference voltage drift.

## 3. Differential Sampling with Integrating Sampler

After calibrating the reference voltage, the amplitude and phase of the AC waveforms provided by a calibrator (Fluke 5720A (Everett, WA, USA)) can be determined by measuring the difference between the AC waveform and the reference waveform. The following section introduces the configuration employed for differential sampling with a commercial DAC. The optimal aperture time for the sampler can be determined by taking into account the transients of the reference waveform, setup time, and voltage range of the sampler. Meanwhile, the derivation of the uncertainty propagation model of DFT is presented. Subsequently, the optimal number of differential measurements is calculated.

### 3.1. System and Setup

**Figure 7:**
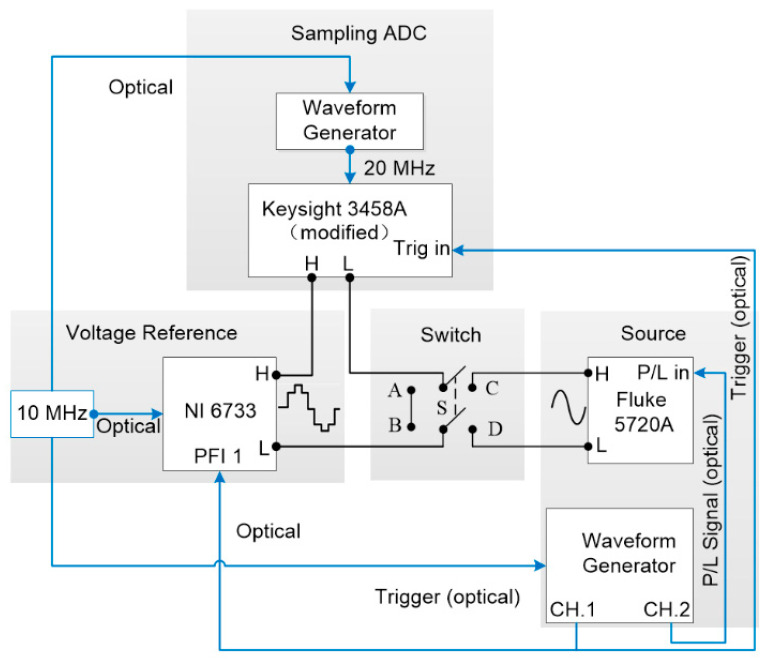
Block diagram of the differential sampling with integrating sampler and a commercial DAC for reference.

The configuration of the differential sampling with a commercial DAC for reference is shown in Figure 7. The differential sampling mode (C, D) and reference voltage calibration mode (A, B) are controlled by a switch (S). The Keysight 3458A is modified to use an external 20 MHz reference clock from the signal generator. The trigger signals of the DAC and sampler are both provided by another signal generator. The signal generators use the same 10 MHz external reference clock for synchronization. All clocks and triggers are connected by optical coupling isolators to eliminate the influence of ground noise. 

The waveform is reconstructed by summing the measured DAC step voltages and the measured difference voltages. The integrating sampler measures the average differential signal over a period and produces one data point per step, and the residual signal after differential sampling contains multiple harmonics. Therefore, we obtain the RMS values of the fundamentals and harmonics by performing a DFT analysis. In this case, the error contributions of the sampler are mainly due to the limited bandwidth and non-zero aperture time. According to [16], the error due to limited bandwidth for the DCV mode of the sampler can be corrected by frequency response compensation, and the error due to the non-zero aperture time is corrected by the transfer function [17]:

(1) $$\alpha_{m}=\sin\left(\frac{m\pi }{N}\cdot r\right)/\frac{m\pi }{N}\cdot r,$$

where *r* is the aperture ratio, which is the ratio of aperture time to duration of DAC step, and *m* is harmonic order, *m* = 0, 1, …, M. When *m* = 0, it is the DC offset, and *m* = 1 is the RMS value of the fundamental.

### 3.2. Aperture Time

Aperture time (defined as *t_i_*) is an important parameter of the sampler. To achieve the highest accuracy and ensure the sampler completes its required setup time, it is necessary to have a delay time (defined as *t_d_*) of at least 30 µs [7,8]. The duration of the DAC step can be defined as

(2) $$t_{s}=\frac{1}{f\cdot N}=t_{i}+2t_{d},$$

where *f* is the frequency of the waveform. Additionally, according to [10], to measure differential voltage in the 100 mV range with a calibrator amplitude of 0.707107 V_RMS_ (corresponding to a 1 V amplitude) and *N* = 20, the aperture ratio should be below 0.6. The selection of aperture time should also avoid the transients of the reference waveform. The delay time of the sampler is sufficient to avoid the transients [18]. Therefore, in the conditions (the calibrator amplitude is 0.707107 V_RMS_ and *N* = 20), the aperture time is limited by the delay time of the sampler when the waveform frequency *f* ≤ 666 Hz and by the voltage range of the sampler when the waveform frequency *f* > 666 Hz.

**Figure 8:**
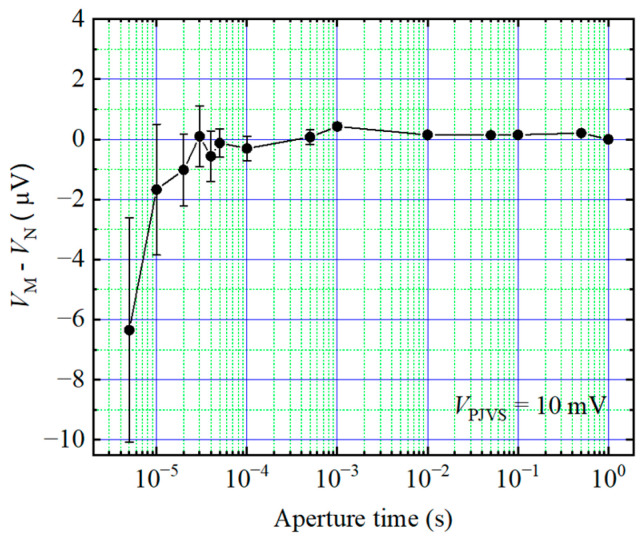
Difference voltage between the measured DC voltage and the nominal voltage at different aperture times. The nominal voltage of the calibrator is 10 mV.

According to the Keysight 3458A user’s guide, a short aperture time results in a smaller number of digits, which affects the measurement accuracy. In addition, Keysight 3458A shows some random gain errors with shorter aperture times [19]. To explore the effect of short aperture time further, the calibrator is set to generate a DC voltage of 10 mV (the voltage of differential sampling was below 10 mV in the experiments in this paper). For 10 mV DC voltage, the sampler range is set to 100 mV. Figure 8 depicts the difference voltage between the measured DC voltage and the nominal voltage (*V*_N_) with different aperture times. The results show that the random gain error of small signals (10 mV) is much smaller than that of large signals (1 V) (as shown in Figure 2) when the aperture time is short. Figure 8 shows the consistency between the measured voltage and the nominal voltage, which is within 10^−6^ orders when the aperture time *t_i_* is equal to or greater than 20 µs (*t_i_* ≥ 20 µs) with small voltages. Therefore, it is important to adjust the aperture time as the waveform frequency increases when using the differential sampling method. This can be achieved by reducing the number of steps to prevent increased uncertainty resulting from a small aperture time. However, it is important to avoid making the number of steps too small, as this can lead to nonlinear effects and increase uncertainty due to larger voltage differences. From (2), with the conditions that the frequency of the calibrator is 50 Hz and the aperture time *t_i_* is greater than 20 µs, the number of steps should be below 250.

### 3.3. Number of Measurement Cycles 

Increasing the number of measurements reduces measurement noise but also increases measuring time. In this section, a theoretical analysis is conducted to determine the cycle of measurements needed to achieve the desired level of uncertainty. This section optimizes the measurement procedure described in the next section.

The RMS measurement uncertainty caused by random noise fluctuations can be represented as

(3) $$u^{2}(V_{\mathrm{rms}})=\sum_{m=0}^{M}{\left(\frac{\partial V_{\mathrm{rms}}}{\partial V_{mc}}\right)}^{2}\cdot u^{2}(V_{mc}).$$

According to the DFT analysis, the uncertainty of the harmonic can be expressed as

(4) $$u^{2}(V_{\left(m,rms\right)})=\frac{4}{N^{2}}\sum_{n=0}^{N-1}\sin^{2}(nmh+\varphi_{m})\cdot u^{2}(v_{n}),$$

where *v_n_* is the voltage of the reconstructed waveform, *N* is the number of steps, *h* = 2π/*N* and *φ_m_* = arctan (*b_m_*/*a_m_*).

Finally, according to (3) and (4), the uncertainty of the RMS amplitude can be derived as

(5) $$\begin{array}{l} u^{2}(V_{\mathrm{rms}})=\frac{4}{N^{2}V_{rms}^{2}}\sum_{m=0}^{M}K_{m}^{2}V_{m}^{2}\cdot \sum_{n=0}^{N-1}\sin^{2}(nmh+\varphi_{m})\cdot u^{2}(v_{n})\\ =\frac{4}{N^{2}V_{\mathrm{rms}}^{2}}\cdot \left[K_{0}^{2}V_{0}^{2},\ldots ,K_{M}^{2}V_{M}^{2}\right]\cdot \left(\begin{array}{ccc} \sin^{2}\theta_{00} & \ldots & \sin^{2}\theta_{0N}\\ \vdots & \ddots & \vdots \\ \sin^{2}\theta_{M0} & \cdots & \sin^{2}\theta_{MN} \end{array}\right)\left(\begin{array}{l} u^{2}(v_{0})\\ \vdots \\ u^{2}(v_{N}) \end{array}\right). \end{array}$$

Equation (5) shows that both the phase and amplitude of harmonics affect the uncertainty of the *V*_RMS_. Assuming that the sampling points are independent of each other and the variance is $\sigma_{x}^{2}$, (6) could be simplified as

(6) $$u^{2}(V_{\mathrm{rms}})=\frac{2}{N}\cdot \sigma_{x}^{2}.$$

Therefore, to obtain the uncertainty of target ($u_{target}^{2}$), the number of measurement cycles should be

(7) $$C=\frac{u^{2}(V_{\mathrm{rms}})}{u_{\mathrm{target}}^{2}}.$$

According to Section 2, the uncertainty due to reference voltage is several parts in 10^7^, and thus, an uncertainty of one part in 10^7^ is expected. The experiments show that the standard deviation of a single time difference measurement is approximately two parts in 10^5^. From (7), a minimum of 4000 consecutive measurement cycles is required. It takes approximately 30 s to measure 4000 cycles continuously; the time is much less than 1000 s (the drift has a significant effect as discussed in Section 2.2). Therefore, any error generated by drift during this period can be ignored.

## 4. Measurements

**Figure 9:**
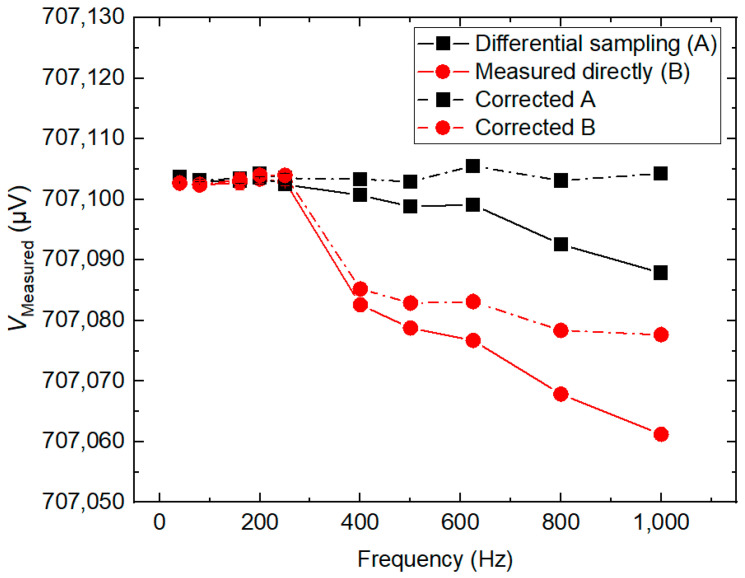
The amplitudes of the fundamental for the calibrator measured directly with an integrating sampler and measured with the differential sampling method in a frequency range of 40 Hz ≤ *f* ≤ 1 kHz with *N* = 20, respectively.

**Figure 10:**
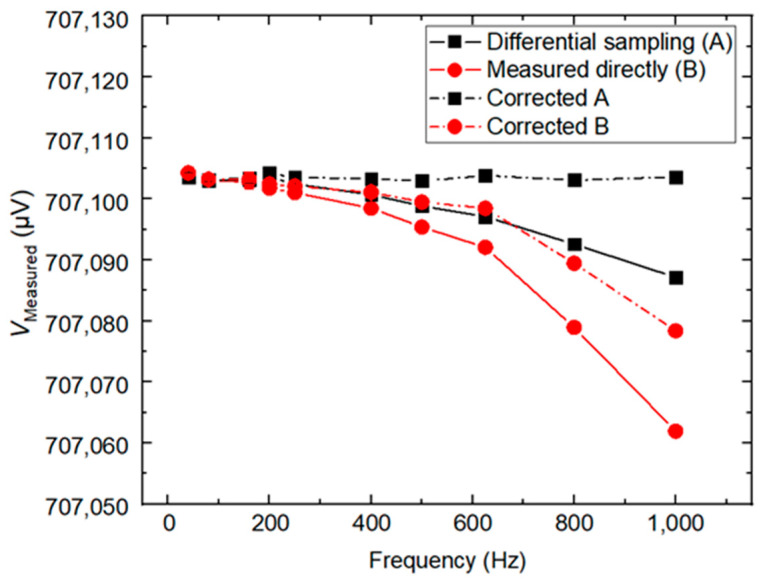
The amplitudes of the fundamental for the calibrator measured directly with an integrating sampler and measured with the differential sampling method in a frequency range of 40 Hz ≤ *f* ≤ 1 kHz with *N* = 8, respectively.

Figure 9 shows the fundamental amplitudes for the calibrator measured directly with an integrating sampler and by the differential sampling method in the frequency range of 40 Hz ≤ *f* ≤ 1 kHz, *V*_Measured_, respectively. The Type A uncertainty is of the order of 0.2 µV/V (the error bars are hidden by the symbols.). In Figure 9, the *V*_Measured_ can be roughly described as a function of *f*, as represented by the solid curves in the figure. To reduce the systematic errors introduced by the sampler input filter, the results were corrected by the method proposed in [16], which is represented by the dotted curves. The calibrator was set to a constant RMS amplitude of V_NOM_ = 0.707107 V_RMS_ with *N* = 20. The comparison between direct and differential sampling measurements shows an agreement within 1 µV/V for frequencies below 100 Hz. Above 200 Hz, the two methods show significant differences, the difference is more than 10 µV/V. Due to the aperture time below 167 µs for frequencies above 200 Hz, resolution is limited to 5.5 digits instead of the maximum 6.5 digits. To verify this difference further, a longer aperture time (change *N* = 20 to *N* = 8) is used to compare the fundamental amplitude of direct sampling and differential sampling at different frequencies. Figure 10 shows the measurements in the frequency range of 40 Hz ≤ *f* ≤ 1 kHz with *N* = 8. Compared with Figure 9, the results of direct measurement have been significantly improved. In Figure 9 and Figure 10, the differential sampling method is more reliable than direct measurement. 

**Figure 11:**
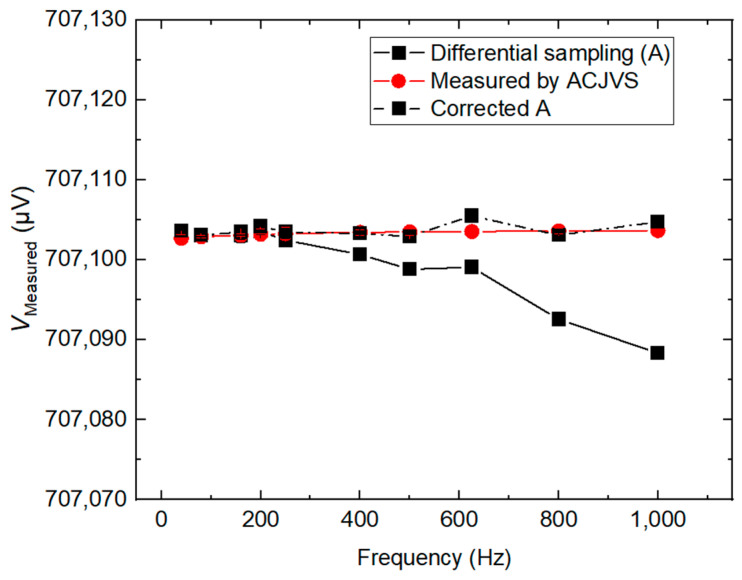
The amplitudes of the fundamental for the calibrator measured with the differential sampling method and ACJVS in the frequency range of 40 Hz ≤ *f* ≤ 1 kHz, respectively.

A comparison with the ACJVS [20] to determine the amplitude of the calibrator at each frequency is shown in Figure 11. The differential sampling system and the ACJVS system are connected in parallel and can be selected from a switch. The measurement of the calibrator is performed alternately by the two systems. The agreement between the differential sampling method and the ACJVS is of the order of 1 µV/V in the frequency range of 40 Hz ≤ *f* ≤ 200 Hz. 

In order to evaluate the reliability and stability of the differential sampling system in this paper, the measurement must be independent of the following measurement parameters:

(a) The aperture time of the sampling voltmeter;
(b) The phase alignment between the calibrator sine wave and the DAC stepwise approximated waveform;
(c) The number of steps of the DAC waveform per period.

A DFT analysis was used to obtain the amplitude and phase of the sinusoidal wave and the first five harmonics. The RMS amplitude of the calibrator was V_NOM_ = 0.707107 V_RMS_ and a frequency of *f* = 50 Hz with *N* = 20 and *r* = 0.6. The results were shown in Table 1 (the phase angle is relative to the sampling window). The harmonic contributions were found to be very small and had a negligible impact on the total RMS value.

**Figure 12:**
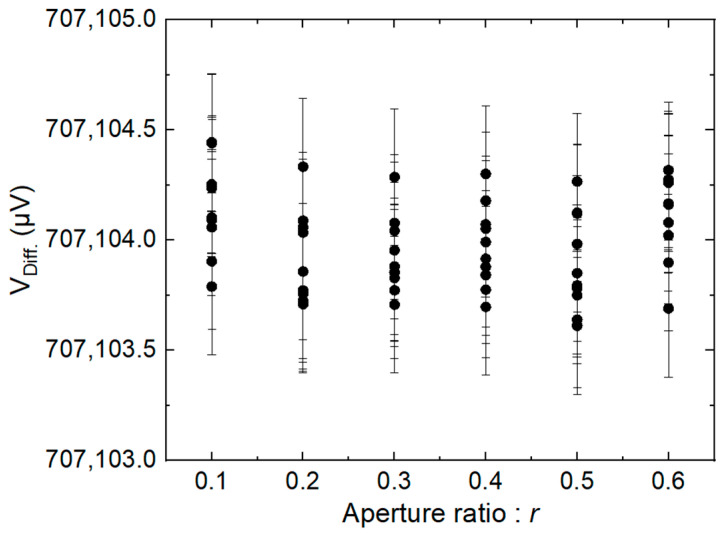
Measurement of the calibrator RMS amplitude versus the aperture ratio of the sampler.

Figure 12 shows the amplitudes of the fundamental for the calibrator measured with the differential method with different aperture ratios of the sampler, where *V*_Diff_. is the RMS amplitude of the fundamental measured with the differential method. The result shows that no significant variation of the calibrator amplitude is observed with aperture ratios changed from 0.1 to 0.6. 

**Figure 13:**
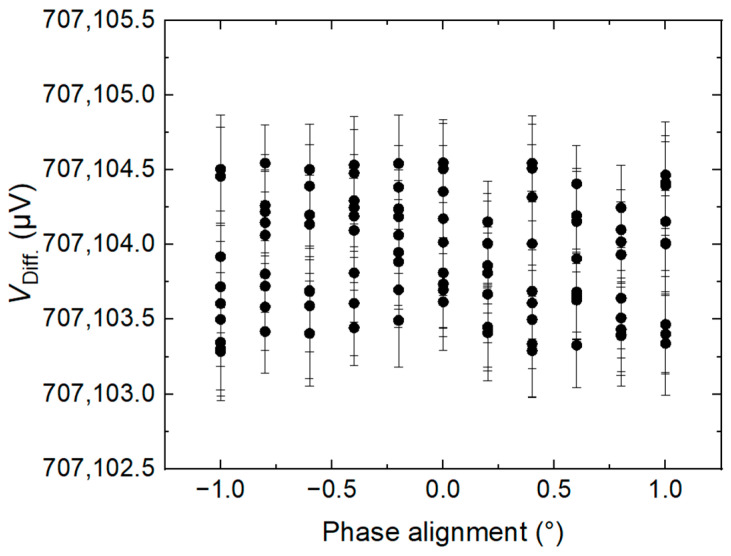
Influence of the phase between the calibrator output and the stepwise approximated waveform on the measured difference at 50 Hz and 0.707107 V_RMS_.

Figure 13 shows the amplitudes of the fundamental measured with the differential method for the calibrator over a range of ±1° variation in phase alignment between the calibrator sine wave and the DAC stepwise waveform. The calibrator’s phase is adjusted by a signal generator, and 0° is defined as the phase resulting in the minimum voltage difference between the DAC stepwise waveform and the AC waveform. The result shows that the measurement system in this paper is independent of the phase alignment between the calibrator sine wave and the DAC stepwise waveform within two degrees. 

**Figure 14:**
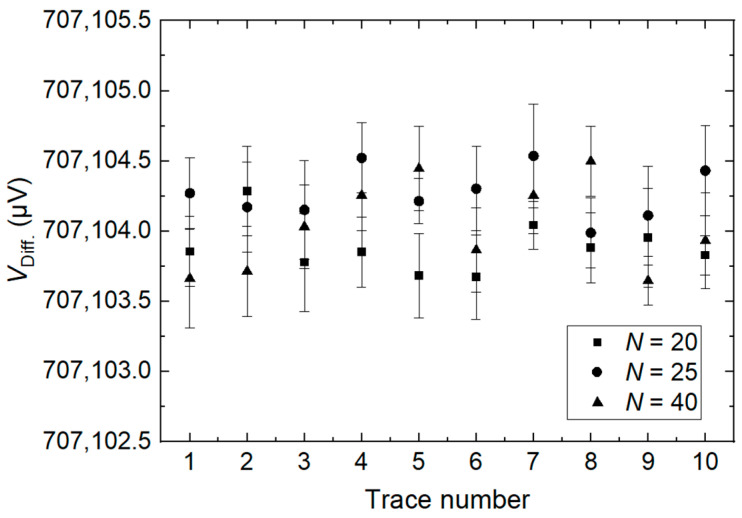
Influence of the number of steps per period used in the DAC waveform on the measured difference voltage to the calibrator output.

Figure 14 shows the amplitudes of the fundamental for the calibrator measured with the differential method with different numbers of DAC steps per period. From the result, there is a negligible variation in the calibrator amplitude when the number of DAC steps per period varies from 20 to 40. This result confirms that the measurement system is independent of the number of DAC steps within the measured uncertainty.

These results show that a commercial-DAC-based differential measurement system has excellent stability and reliability. 

## 5. Uncertainty Evaluation

### 5.1. Evaluation of the Sampler

**Figure 15:**
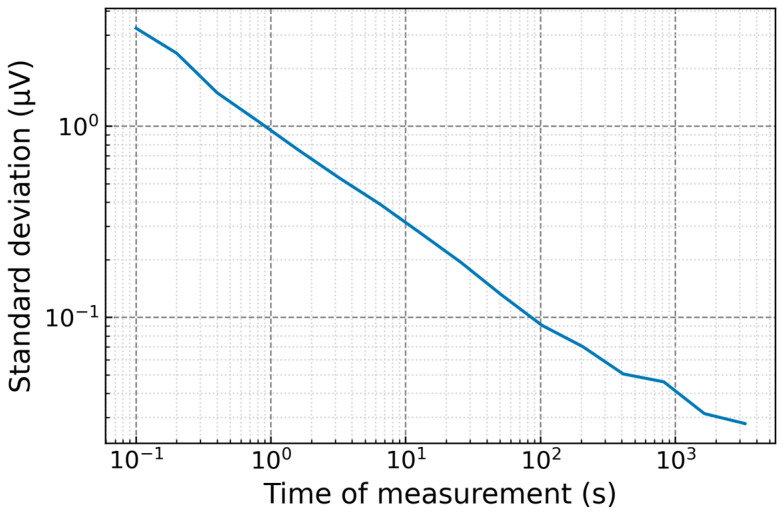
The Allan deviation analysis of short-circuit voltage of the sampler.

The short-circuit voltage of the sampler was measured under the same conditions as the reference voltage measurements described in Section 2. The Allan analysis of the short-circuit voltage of the sampler is shown in Figure 15. Based on the Allan deviation analysis, the standard deviation due to sampler noise was evaluated to be less than 0.2 µV, and the uncertainty of measurement due to sampler noise is about 0.1 µV/V following a rectangular distribution.

In order to evaluate the uncertainty of the gain correction error for the sampler, the sampler was calibrated using a PJVS system as described in [21]. Based on the uncertainty evaluation of the parameter for the least-square fitting, the relative standard uncertainty of the slope is 0.051 µV/V for a 1 V range and 5.4 µV/V for a 100 mV range. As the amplitude at the input of the sampler is reduced to less than 10 mV during differential voltage measurements, the uncertainty of measurement due to the gain correction error is about 0.06 µV/V following a rectangular distribution. 

According to the specification of the Keysight 3458A and the measurements in [14,22], the common-mode error of the sampler is 9.93 µV_RMS_ with 100 V_RMS_ of the 50 Hz signal. The uncertainty due to the common-mode error of the sampler was calculated to be 0.04 µV/V with a rectangular distribution. Furthermore, the effect of this limited bandwidth (150 kHz) of the sampler cannot be negligible. As [13,23] shows, the uncertainty due to the limited bandwidth is about 0.1 µV/V at the frequency of 50 Hz.

### 5.2. Evaluation of the DAC and the Calibrator

As described in Section 2, the uncertainty due to the noise is about 0.20 µV/V with a Gaussian distribution. There is an error of approximately 0.4 µV in the measurement when comparing the dynamic method to the static method. The uncertainty due to the methodical error is about 0.2 µV/V with a rectangular distribution. According to Figure 5, as the reference voltage is calibrated within 1000 s, the uncertainty due to the drift of the reference waveform is less than 0.01 µV/V. The noise is the major contributor to Type A uncertainty. The temperature coefficient error of the DAC described in the manufacturer’s specification is ±0.6 ppm/°C. All experiments in this paper were performed in a constant temperature laboratory, and the range of temperature was within ±0.1 °C in half an hour. The uncertainty due to the temperature coefficient is lower than 0.04 µV/V with a U-shaped distribution.

The measurements are also affected by the drift of the calibrator and the relative error in phase locking between the reference waveform and the calibrator. The drift of the calibrator is reported to be about 0.3 µV/V/min [24]. The uncertainty was calculated to be 0.3 µV/V with a Gaussian distribution. The uncertainty of phase error was determined by the maximum deviation of about 0.4 µV from the mean value in Figure 13 with a rectangular distribution. The uncertainty due to phase error was calculated to be 0.2 µV/V. In addition, the noise of the calibrator also contributes to system noise. Long-term Allan analyses show that the uncertainty due to calibrator noise was evaluated to be less than 0.2 µV/V with a rectangular distribution.

An uncertainty budget for differential sampling measurement using a commercial DAC for reference at 50 Hz and 1 V amplitude is presented in Table 2.

## 6. Conclusions

This study has successfully introduced and validated a differential sampling methodology utilizing a commercial digital-to-analog converter (DAC) as a reference for the precise calibration of AC signals. By converting AC signals into a combination of large DC and smaller AC components, we utilize the superior performance of DCV mode and smaller gain error in the small signal of the Keysight 3458A. By adopting a static over a dynamic measurement approach and carefully selecting the optimal aperture ratio, we have optimized the accuracy of reference voltage measurements. Additionally, we developed an uncertainty propagation model based on discrete Fourier transform (DFT), essential for designing the measurement process and evaluating result uncertainty. This method offers a cost-effective and straightforward alternative to PJVS-based systems. Additionally, the differential sampling method shows that it has obvious advantages over direct measurement by the integrating sampler as the frequency increases. Experimental comparisons between the differential sampling method and the ACJVS have demonstrated an agreement of the order of 1 µV/V in a frequency range of 40 Hz to 200 Hz, and this result may hold up to 1 kHz after correction. In the case of the fundamental, the expanded uncertainty (*k* = 2) was evaluated to be approximately 1 part in 10^6^ for a 50 Hz sine wave with a 0.707107 V_RMS_ generated by a calibrator. Repeated experiments have demonstrated the robustness and parameter independence of this approach, providing the way for improved precision and efficiency in AC waveform calibration.

## Figures and Tables

**Table 1:** The RMS amplitude and phase of the fundamental and the first five harmonics of the sine wave.

	RMS Amplitude (µV)	Phase (deg)
1*f*	707,104.29 ± 0.32	−0.006 ± 0.0001
2*f*	29.15 ± 0.03	115.31 ± 0.10
3*f*	6.52 ± 0.03	−83.67 ± 0.45
4*f*	0.56 ± 0.01	−96.10 ± 2.68
5*f*	2.71 ± 0.01	−119.20 ± 0.96
DC off.	126.55 ± 0.97	

**Table 2:** Uncertainty budget for the differential calibration of 0.707107 Vrms at 50 Hz.

Source of Uncertainty	Component	Type	Uncertainty (μV/V)
Reference voltage (DAC)	Noise of step	A	0.20
	Methodical error	B	0.20
	Drift	B	0.01
	Temperature coefficient	B	0.04
Calibrator	Noise of calibrator	A	0.20
	Drift	B	0.30
	Phase error	B	0.20
Sampling voltmeter	Noise of sampler	A	0.10
	Common-mode rejection ratio	B	0.04
	Gain correction error	B	0.06
	limited bandwidth	B	0.10
Combined standard uncertainty			0.53
Expanded uncertainty (*k* = 2)			1.06

## Data Availability

The experimental data presented in this study are available upon reasonable request from the corresponding author.
